# Differential action of pro-angiogenic and anti-angiogenic components of Danhong injection in ischemic vascular disease or tumor models

**DOI:** 10.1186/s13020-021-00557-5

**Published:** 2022-01-04

**Authors:** Shuang He, Rongrong Chen, Li Peng, Zhenzuo Jiang, Haixin Liu, Zihao Chen, Tiechan Zhao, John Owoicho Orgah, Jie Ren, Peng Zhang, Yuefei Wang, Xiumei Gao, Yan Zhu

**Affiliations:** 1 State Key Laboratory of Component-based Chinese Medicine, Tianjin University of Traditional Chinese Medicine, 10 Poyanghu Road, Jinghai District, Tianjin, 301617 China; 2 Research and Development Center of Traditional Chinese Medicine, Tianjin International Joint Academy of Biomedicine, TEDA, 220 Dongting Road, Tianjin, 300457 China

**Keywords:** Danhong injection, Angiogenesis, Bi-directional therapy, Pro-angiogenic, Anti-angiogenic

## Abstract

**Objective:**

We investigate the chemical basis and mechanism of angiogenesis regulation by a multicomponent Chinese medicine Danhong injection (DHI).

**Methods:**

DHI was fractionated and screened for angiogenesis activities by in vitro tube formation and migration assays. The composition of DHI components was determined by UPLC. The effects of the main active monomers on angiogenesis-related gene and protein expression in endothelial cells were determined by qPCR and Western blotting analyses. Mouse hind limb ischemia and tumor implant models were used to verify the angiogenesis effects in vivo by Laser Doppler and bioluminescent imaging, respectively.

**Results:**

Two distinct chemical components, one promoting (pro-angiogenic, PAC) and the other inhibiting (anti-angiogenic, AAC) angiogenesis, were identified in DHI. PAC enhanced angiogenesis and improved recovery of ischemic limb perfusion while AAC reduced Lewis lung carcinoma growth in vivo in VEGFR-2-Luc mice. Among the PAC or AAC monomers, caffeic acid and rosmarinic acid upregulated TSP1 expression and downregulated KDR and PECAM expression. Caffeic acid and rosmarinic acid significantly decreased while protocatechuic aldehyde increased CXCR4 expression, which are consistent with their differential effects on EC migration.

**Conclusions:**

DHI is capable of bi-directional regulation of angiogenesis in disease-specific manner. The pro-angiogenesis activity of DHI promotes the repair of ischemic vascular injury, whereas the anti-angiogenesis activity inhibits tumor growth. The active pro- and anti-angiogenesis activities are composed of unique chemical combinations that differentially regulate angiogenesis-related gene networks.

## Introduction

Angiogenesis is a fundamental process of normal development, including various pathophysiologies such as ischemic heart disease, atherosclerosis, limb disease, and cancer [1]. Inadequate angiogenesis can lead to the disorder of ulcer healing and myocardial infarction. Angiogenesis has the potential to rescue early ischemic myocardium and is essential for long-term left ventricular remodeling to prevent heart failure [2, 3]. In contrast, many pathological conditions, such as cancer and diabetic retinopathy, are characterized by abnormal vascular proliferation. Anti-angiogenic therapy can reprogram the tumor immune microenvironment and prune the blood vessel, which is necessary for tumor growth and metastasis [4]. Therefore, inhibition of angiogenesis has become a common target of many cancer chemo-preventive agents [5]. Nowadays, more than 10 approved anti-angiogenic tyrosine kinase inhibitors for treating patients with advanced cancers [6].

Traditional Chinese medicine (TCM) and other herbal products are a rich source of angiogenesis modulating agents. For example, Ginseng extract has a bi-directional regulation of angiogenesis, which promotes wound healing and inhibits tumor. Those effects were found to be attributable to two opposing ginsenosides, Rg1 and Rb1 [7]. Our analysis showed that many phytoestrogens are capable of either promote or inhibit angiogenesis [8]. A pharmacological review of TCM effect on angiogenesis showed that tanshinones inhibited angiogenesis, whereas salvianolic acid promoted angiogenesis [9]. In addition, rosmarinic acid effectively suppressed tumor growth by regulating the secretion of cytokines associated with inflammation and angiogenesis [10].

Danhong Injection (DHI), a Chinese patent medicine consisting of extracts from *Radix Salviae miltiorrhizae* and *Flos Carthami tinctorii*, has the actions of activating blood circulation and removing stasis. DHI is mainly used for the treatment of cardiovascular and cerebrovascular diseases in the clinics in China. The identified pharmacological properties of DHI include anti-inflammatory, antioxidant, anticoagulatory, hypolipidemic effect, antiapoptotic effect, vasodilator, and angiogenesis-promotor [11–16]. We and others have quantified twenty-three primary metabolites and seven polyphenolic acids in DHI [17]. Other researchers identified a total of sixty-three compounds in DHI, including thirty-three phenolic acids, six organic acids, six flavonoid O-glycosides, five amino acids, four iridoid glycosides, three nucleosides, and two C-glycosyl quinochalcones [18]. DHI increased endothelium-dependent vasorelaxation through the prostacyclin/cyclooxygenase-2 pathway in rat aorta [19] and DHI could reduce vascular remodeling and up-regulates the Kallikrein-kinin system in spontaneously hypertensive rats to lower blood pressure [20]. DHI protected rat cardiomyocyte injury induced by excessive arginine vasopressin and significantly reduced the damage of primary rat neuronal cells and rat cardiomyocytes [21]. DHI promoted the recovery of motor deficits after stroke via parkin-enhanced mitochondrial function and reversed cardiac abnormality in brain-heart syndrome via β-adrenergic receptor signaling [22, 23].

We have shown previously that DHI accelerated recovery from peripheral arterial disease in Type 2 diabetic mice by coordinated activation of VEGF/VEGFR-2 and PPARdelta pathways [24] and this action was attributable to a combined 4 active compounds in DHI [25]. In addition, DHI ameliorated cardiac dysfunction and ventricular remodeling after myocardial infarction by anti-TGF-beta-mediated fibrosis and promoting angiogenesis [26]. But considering that angiogenesis is a double-edged sword and there are Chinese medicines that contain both pro-angiogenic and anti-angiogenic components. DHI as a compound preparation contains many ingredients, and there should also be a phenomenon that DHI contains both pro-angiogenic and anti-angiogenic components. However, the regulation effect of different components in DHI on angiogenesis has not been specifically studied. Therefore, the purpose of this work is to resolve whether the components in DHI has a two-way regulating effect on angiogenesis.

## Methods

### Identification of PAC and AAC components of DHI by chromatography

DHI (product Approval Number Z20026866) was provided by Shandong Buchang Pharmaceutical Co. Ltd. (Heze, China). DHI was extracted with dichloromethane and chloroform in sequence according to the polarity. Chromatographical identification of DHI fractions were performed by an ultra-performance liquid chromatography system (Waters Corp., Milford, MA, USA) equipped with a diode array detector, a column oven, an automatic sampler, and a binary gradient solvent pump as previously reported [27]. We performed chromatography on an ACQUITY UPLC HSS T3 column (2.1 × 100 mm, 1.8 μm) at 40 °C. The mobile phase consisted of 0.1% formic acid aqueous solution (A) and acetonitrile (B). The gradient program was as following: 0–7 min, 3–19% B; 7–13 min, 19% B; 13–18 min, 19–25% B; 18–25 min, 25–90% B; 25–30 min, 90% B. We set the flow rate of the mobile phase to 0.4 mL/min, and the injection volume was 2 µL at the detection wavelengths of 254 and 286 nm.

### Cell culture

EA.hy926 cells (Shanghai Cell Bank, Type Culture Collection Committee, Chinese Academy of Sciences) were cultured in DMEM (Gibco, USA) supplemented with 10% fetal bovine serum (FBS) (Gibco, USA), 100 U/mL penicillin, and 100 µg/mL streptomycin (Hyclone, Thermo Scientific, USA) and were grown in an incubator containing 5% CO_2_ at 37 °C. When reached the desired confluence, cells were passaged by detaching with 0.25% trypsin-EDTA (Gibco, USA). Precisely weigh several milligrams of the extract from each layer of the extract of DHI, use DMSO as the solvent, uniformly prepare the mother liquor concentration of 100 mg/mL, and dilute to the working concentration with cell culture medium.

### Cell toxicity

When EA.hy926 cells were grown to approximately 80% confluences. The effect of PAC or AAC on cell toxicity was evaluated by using CCK-8 kit. In brief, EA.hy926 cells were seeded in 96-well plate at a density of 5 × 10^3^ cells/well and incubated at 37 °C for 24 h. Then, the cells were treated with various concentrations of PAC or AAC. After 48 h incubation, 10 µL CCK-8 solution was added to the wells, and continued for another 2 h incubation. The resulting color was assayed at 450 nm using FlexStation® 3 (Molecular Devices, Emax, Sunnyvale, CA).

### Cell migration assay

Cell migration was measured in two different assays. EA.hy926 cells were seeded into 96-well plates at a density of 2 × 10^4^ cells per well. We stained the cells with 1 µg/mL Hoechst 33,342 (Molecular Probes, USA) at 37 °C for 30 min after 24 h of serum deprivation. The cell monolayer was scraped in a straight line to create a ‘‘scratch’’ with a 200 µl pipet tip, rinsed twice with PBS to remove the debris and then replaced in 100 µl experimental DMEM containing 1% FBS and PAC (50 µg/mL), AAC (25 µg/mL), or vehicle. The plate was inserted into an incubator at 37 °C for 12 h. Images were captured at 0, 4, 8, and 12 h after injury using a High Content Analysis (HCA) microplate imager (Operetta, PerkinElmer, USA) at ×10 magnification. The cell migration was quantified using the line selection tool in ImageJ software by tracing the wound margin of two defined positions in each image. The distance of each scratch closure was calculated by comparing the images from time 0 to the next time point. The oris™ assay was performed in an optically clear 96-well black skirted fluorescence microplate, according to the manufacturer’s instruction [28]. Briefly, cells were seeded and allowed to attach and then spread in the presence of a silicon stopper. Subsequently, the plug was removed, and the central cell-free region was found to be surrounded by monolayer cells that support migration. We cultured the EA.hy926 cells in DMEM containing 10% FBS, and then the cells were plated on orisTM cell migration-collagen I coated plates containing cell seeding stoppers. The cells were grown for 16 to 20 h before the stopper was manually removed. The cells were washed, and then the medium was replaced with DMEM containing 1% FBS, and the indicated concentrations of VEGF, PAC, AAC, or vehicle. After incubation with the compound for 12 h, the media were removed, and the cells were stained with Calcein AM for 30 min at 37 ℃. After washing twice with PBS, images were captured on the Operetta HCA imager. Three technical replicates were done per experiment, and three independent experiments were performed.

### In vitro tube formation assay

We performed the Tube formation assay following a procedure by Michaud [29]. We coated the 96-well plates with a basement membrane matrix (BD Biosciences, USA). EA.hy926 cells were incubated in a drug-containing medium at a density of 1.5 × 10^4^ cells/well. At the end of the 12 h of incubation, the cells were stained with 1 µM calcein-AM for 30 min. Nine fields of view were randomly selected in each well. Imaging was performed using the Operetta HCA imager, which analyzes the ability of tube formation by calculating the number of cellular networks. Three technical replicates were done per experiment, and three independent experiments were performed.

### Real-time quantitative PCR analysis

The angiogenesis-related genes were examined by the real-time quantitative PCR in cultured EA.hy926 cells treated with PAC, AAC, or vehicle and ischemic gastrocnemius muscle. Total RNA samples were extracted from ischemic muscle or ECs using TriQuick Reagent (Solarbio, Beijing, China), followed by reverse transcription of the RNA samples into complementary DNA using the Transcriptor First Strand cDNA Synthesis Kit (Roche, Germany) in accordance with the manufacturer’s protocol.

The resulting cDNA was used as a template for real-time polymerase chain reaction amplification. SYBR Green Master Mix reagent was used for quantitative PCR, and GAPDH was used as an internal control to quantify the level of angiogenesis-related genes. C1000TM Thermal Cycler Sequence Detection System (BIO-RAD, USA) was used to perform the amplification and analysis. Samples were compared using a relative CT method. The fold increase or decrease was determined relative to a blank control after normalizing to a housekeeping gene using 2^-∆∆CT^, GAPDH.

### Preparation of standard and sample solutions

Preparing the standard stock solutions of the sixteen standards of DHI in 50% methanol aqueous solution with ultrasonic mixing. For sample stock solution, 0.107 g PAC and 0.125 g AAC were placed in a 25 mL volumetric flask, respectively, with 50% methanol aqueous solution and methanol volume to the scale. It was ultrasonically mixed completely and cleared by centrifugation.

### Animals

Homozygous VEGFR-2-Luc male mice [30] were obtained from three transgenic breeding colonies that were maintained in a specific pathogen-free animal lab of TJAB. All the experiments were reviewed and approved by the TJAB Animal Experimental Ethics Committee (TJAB-JY-2011-002) and conducted in accord with the guidelines for the use of animal experiments at Tianjin University of Traditional Chinese Medicine. Animals were maintained on a normal diet and allowed to adapt to the environment for 72 h before the experiment. A constant temperature of 22 °C was provided in a 12 h light/dark cycle, food and water were provided freely.

### Murine hind-limb ischemic model

Mice were anesthetized with isoflurane, and unilateral hind-limb ischemia was induced as previously described [24]. The entire femoral artery and vein of the right hind limb were exposed, and the exposed vessels were ligated at their proximal and distal ends. Both vessels were excised in the middle. The intact perfused contralateral limb of each mouse was used as an internal control. After hind-limb ischemia, saline, simvastatin, DHI, PAC, or AAC were administrated daily for 21days. The dosage of the PAC per mouse was 13.8 mg/kg, and the dosage of the AAC was 69 mg/kg. The preparation method of PAC and AAC in vivo: first dissolve with 10% volume of absolute ethanol, then add 90% volume of normal saline to mix, intraperitoneal injection, once a day. LDPI system was used to perform a continuous, non-invasive assessment of ischemic limb microvascular perfusion. The ratio of perfusion in the ischemic limb to perfusion in the healthy limb was monitored periodically as an indicator of recovery of hind limb perfusion.

### Murine tumor implant model

VEGFR2-Luc mice were used to establish the Lewis lung carcinoma (LLC) animal model. 100 µl saline containing 2.5 × 10^5^ of LLC cells was injected subcutaneously into the axilla of both forelimbs of the VEGFR2-Luc mice. The day after tumor implantation, the animals were randomized into control, DHI, PAC, and AAC-treatment groups. At 11 days after treatment, protruding tumors were found at the injection sites. Using a caliper, the tumor size was measured every three days. At the end of the treatment, mice were sacrificed, and the tumor was removed and weighted.

### In vivo bioluminescent imaging

IVIS1 Lumina K Series III system (PerkinElmer) was used to provide real-time, rapid in vivo imaging enabling the acquisition of biologically relevant events within milliseconds. Mice were anesthetized with isoflurane, and then 150 mg/kg of D-luciferin (PerkinElmer) was injected intraperitoneally for each mouse. The optical signal intensity of VEGFR-2-Luc mice was obtained 5 min after D-luciferin administration. The regions of interest (ROI) from the displayed images were identified on the ischemic sites or tumor sites using LivingImage® software and quantified as photons per second (p/s).

### Western blotting

EA.hy926 cells were treated with different drugs in 10 cm dishes for 24 h. We lysed the cells with a lysis buffer containing protease inhibitors. The lysate was transferred to a 1.5 mL centrifuge tube and incubated on ice for 30 min with a vortex. Total protein was obtained by centrifugation at 12,000 × g for 30 min at 4 °C, and the protein concentration was measured by a BCA kit. After the addition of the loading buffer, the samples were boiled and separated using sodium dodecyl sulfate-polyacrylamide gel electrophoresis (SDS-PAGE). The proteins were to (polyvinylidene difluoride) membranes and then blocked ( 5% skim milk) for 2 h at room temperature. Thereafter, it was incubated with antibodies against CXCR4 or GAPDH overnight on a 4 °C shaker. The membranes were washed trice with PBST and then incubated with the secondary antibodies for 1 h at room temperature in the dark. The membranes were washed trice with PBST, and then the chemiluminescence signals were detected by ECL Plus detection system and exposure to X-ray film to produce bands within the linear range.

### Statistical analysis

Data analyses were performed with SPSS16.0 statistical software. The results were shown as mean ± SD. Statistical significance was assessed by unpaired Student’s *t-test* or by analysis of variance test for comparison between multiple groups. A P-value of < 0.05 was taken as statistically significant.

## Results

### Identification of the composition of PAC and AAC by UPLC

**Fig. 1:**
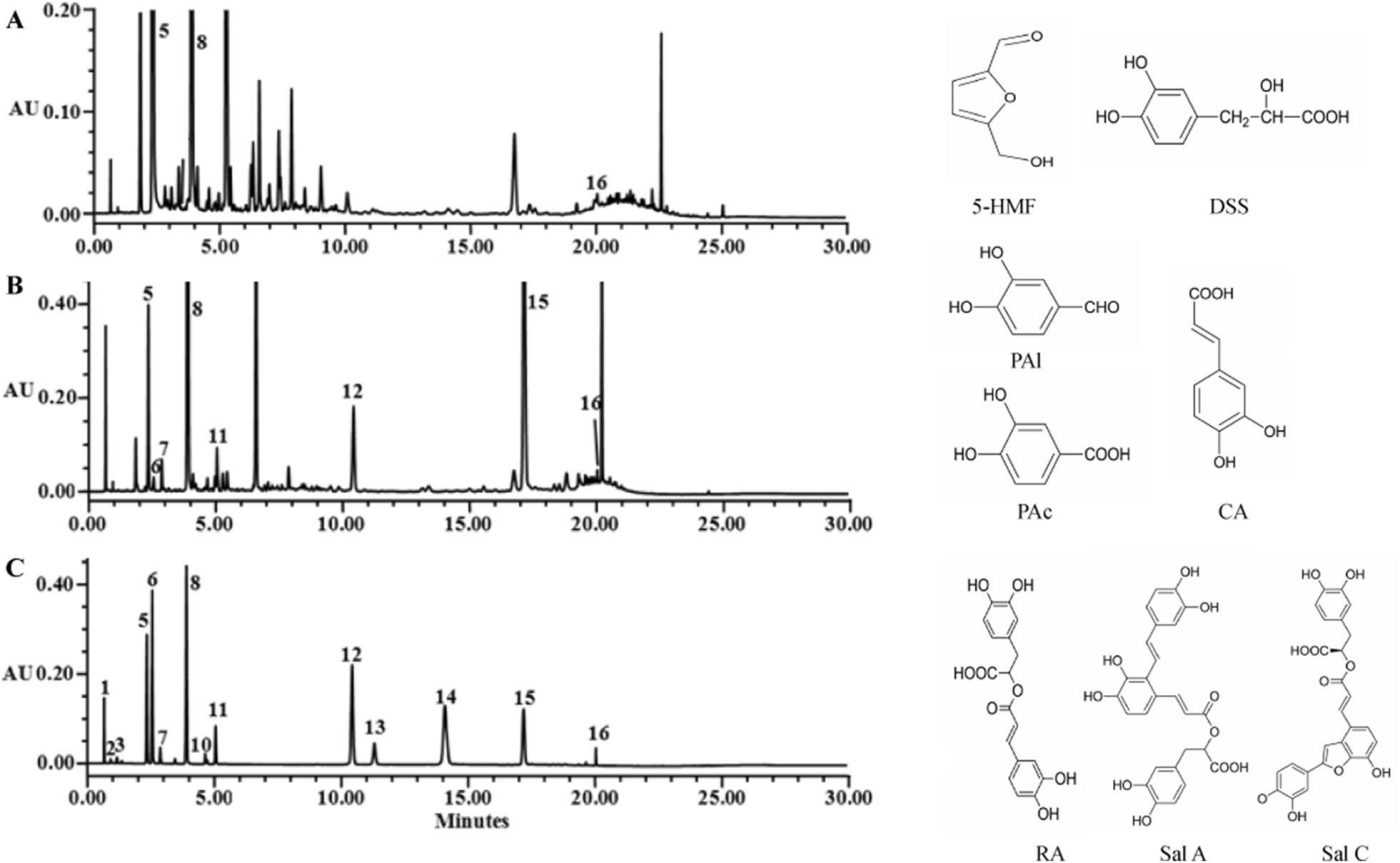
UPLC chromatograms of PAC (**A**), AAC (**B**), and standard mixture (**C**). Peaks: 1. cytidine, 2. uridine, 3. adenosine, 5. 5-hydroxymethyl-2-furfural (5-HMF), 6. Danshensu (DSS), 7. protocatechuic acid (PAC), 8. protocatechuic aldehyde (PAl), 10. hydroxysafflor yellow A (HSYA), 11. caffeic acid (CA), 12. rosmarinic acid (RA), 13. lithospermic acid (LA), 14. salvianolic acid B (Sal B), 15. salvianolic acid A (Sal A) and 16. salvianolic acid C (Sal C)

We have previously constructed a fraction library of DHI and screened several pharmacological activities [19, 27]. Here, a tube-formation assay was used to screen the angiogenic activity of the DHI fractions in vascular endothelial cells. We not only identified a fraction that clearly promoted angiogenic activity (F^#^II), but surprisingly, another fraction that significantly inhibited angiogenic activity (F^#^III). They were assigned as pro-angiogenesis component (PAC) and anti-angiogenesis component (AAC) respectively, for the follow-up investigation below. The UPLC chromatograms of PAC and AAC along with the standard are shown in Fig. 1 and the quantitation (total percentage) of the compounds in PAC and AAC are shown in Table 1.

**Table 1:** The total percentage of the compounds in PAC and AAC

	5-HMF	DDS	PAc	PAl	CA	RA	Sal A	Sal C	Total content (%)
PAC	6.275	–	–	4.986	–	–	–	0.0402	11.43
AAC	0.519	0.595	0.408	7.35	0.269	2.064	8.769	0.065	20.4

*PAC* pro-angiogenesis component, *AAC* anti-angiogenesis component, *5-HMF* 5-hydroxymethyl-2-furfural, *DDS* Danshensu, *PAc* protocatechuic acid, *PAl* protocatechuic aldehyde, *CA* caffeic acid, *RA* rosmarinic acid, *Sal A* salvianolic acid A, *Sal C* salvianolic acid C

### Evaluating the cell toxicity of PAC and AAC by CCK-8 assay

**Fig. 2:**
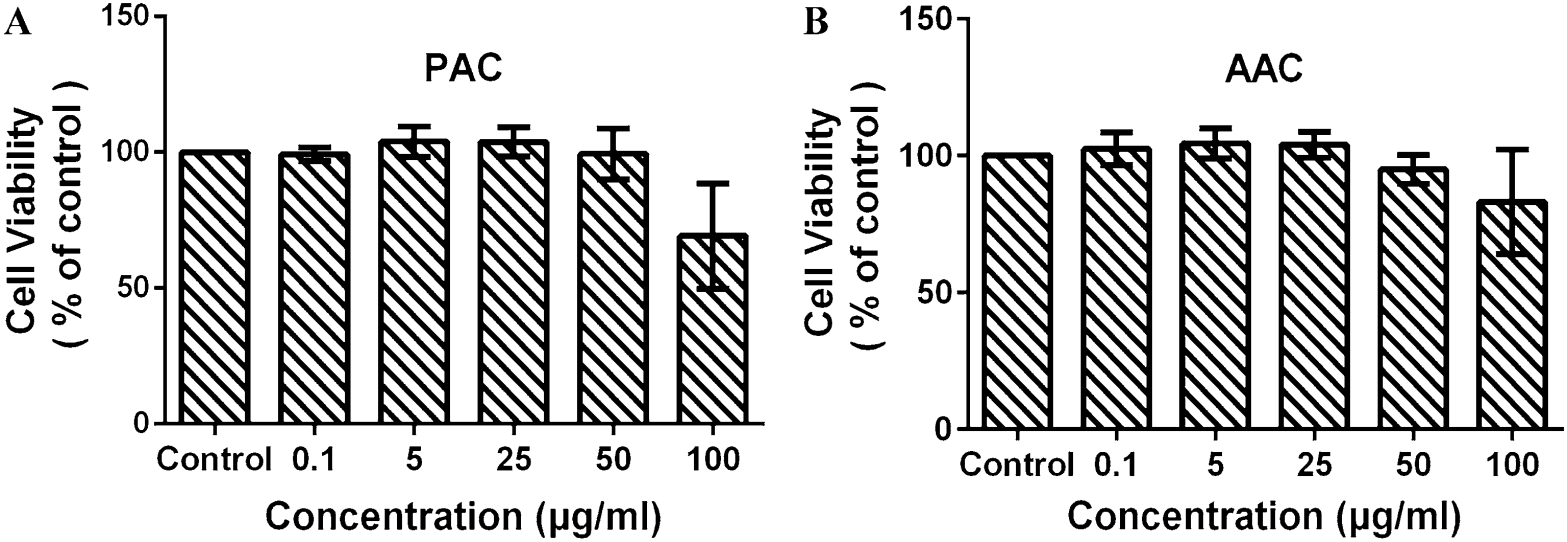
PAC and AAC displayed no toxicity on endothelial cell. **A** EA.hy926 cells toxicity was determined by a CCK-8 assay under different concentrations of PAC. **B** EA.hy926 cells toxicity was determined by CCK-8 assay under different concentrations of AAC. Data are presented as mean ± SD. n = 3

The toxicity of PAC or AAC after treatment in the EA.hy926 cells was determined by Cell Counting Kit-8 assay. The dose of PAC or AAC we chose in the tube formation has no toxicity on the cells (see Fig. 2).

### PAC and AAC impacted oppositely on angiogenesis in endothelial cell in vitro

**Fig. 3:**
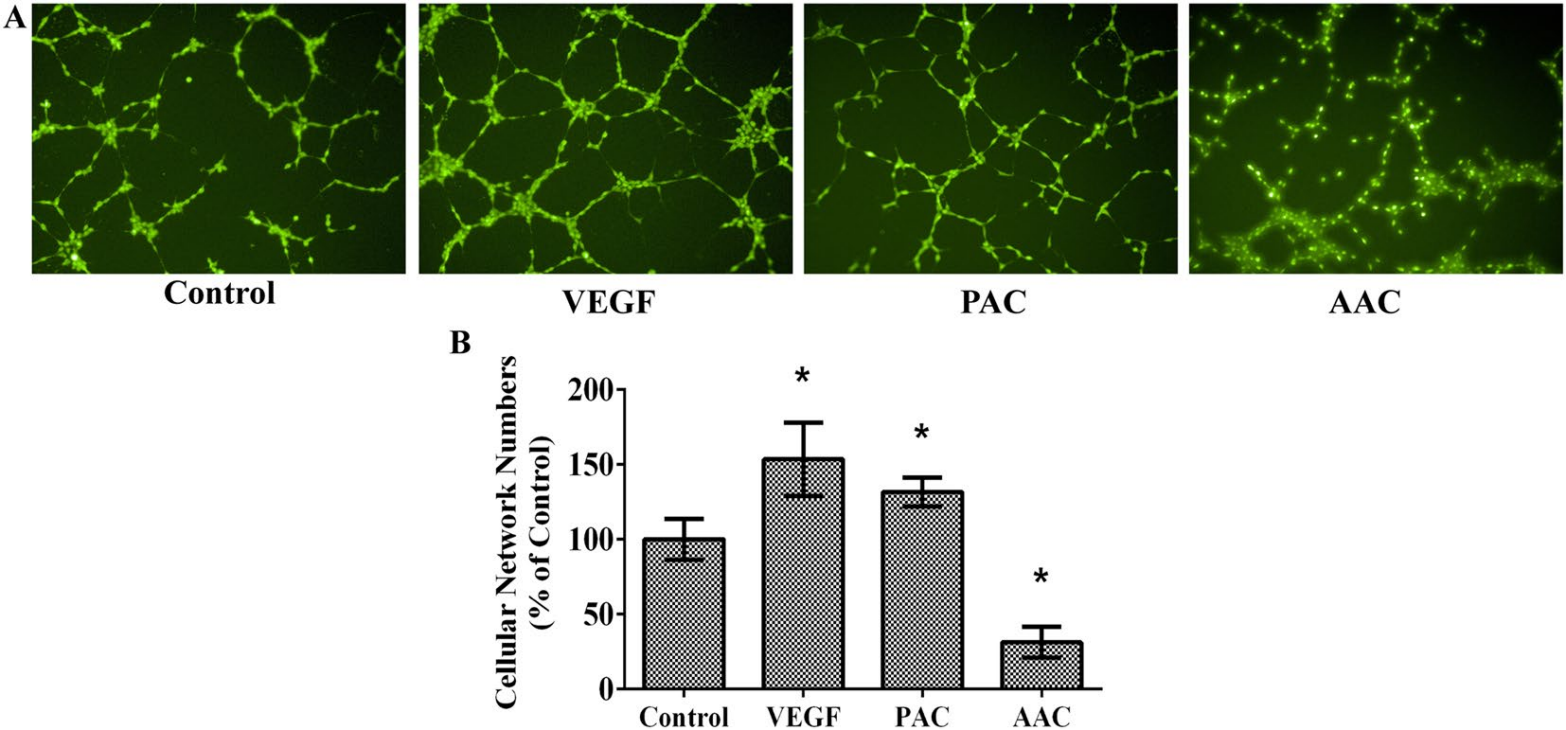
PAC and AAC displayed bi-directional effects on endothelial cell angiogenesis in tube formation assay. **A** Representative microscopy image of tube formation in EA.hy926 cells. **B** Quantitation of *in vitro* tube formation activity. After incubating for 12 h, numbers of the cellular network were counted in multiple (n = 3) views. The average percentage was calculated. VEGF and PAC promoted tube formation, whereas AAC inhibited tube formation in EA.hy926 cells. Data are presented as mean ± SD. n = 3. ^*^P < 0.05, compared with the control group

The classic assay for angiogenesis is to determine the ability of endothelial cells in vitro to form capillary-like structures with a lumen. Our results showed that similar to VEGF (50ng/mL), PAC promoted, but AAC inhibited the tube formation ability in vitro compared with the control group (Fig. 3).

### PAC and AAC affected endothelial cell migration differently

**Fig. 4:**
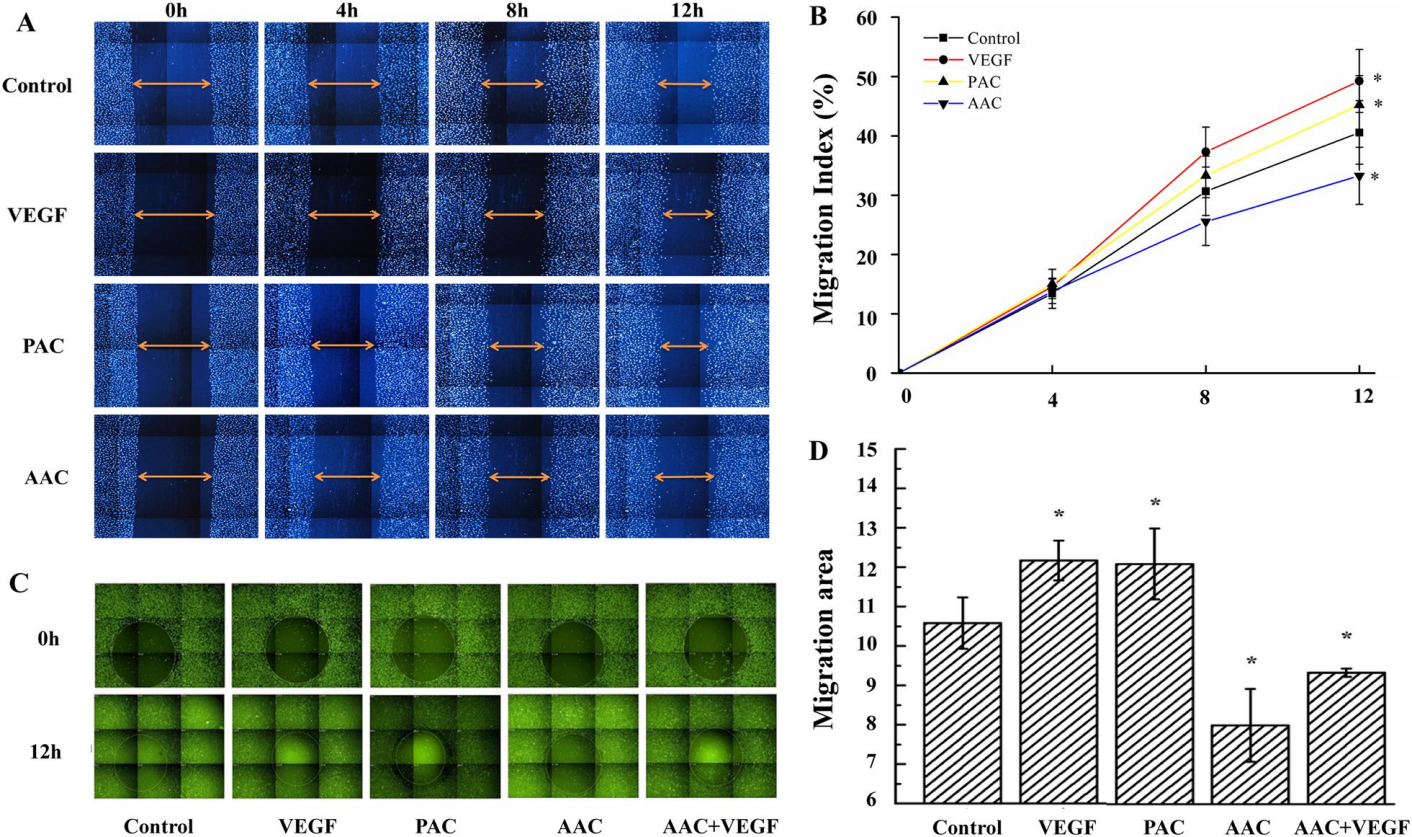
PAC and AAC affected endothelial cell migration differently. **A** Representative images of the wound-healing assay in EA.hy926 cells. **B** Quantitation of the migration distance. PAC or VEGF promoted cell migration while AAC inhibited the cell migration after 12 h. **C** Representative images of the Oris™ Cell Migration Assay in EA.hy926 cells. **D** Analysis of ECs-covered area after 12 h incubation or immediately at the stopper removal (pre-migration, 0 h). The same location for all wells was set aside as a detection zone (Green Square), and the cell inhabited area was determined under the indicated conditions. VEGF or PAC promoted cell migration, while AAC inhibited cell migration after 12 h. AAC also reversed the cell migration effect of VEGF, leading to an ultimate inhibition of endothelial cell migration. n = 3. Data are presented as mean ± SD. *P < 0.05, compared with the control group

The effect of PAC and AAC on endothelial cell migration was determined by a wound-healing assay and Oris™ Cell Migration Assay. Similar to VEGF (50 ng/mL) control, PAC significantly increased, but AAC significantly decreased the migration rate of EA.hy926 cells after 12 h (Fig. 4).

### PAC, but not AAC, improved perfusion of ischemic limb and enhanced angiogenesis in VEGFR-2-luc mice by increasing the expression of FGF-2, CXCR4 and PECAM

**Fig. 5:**
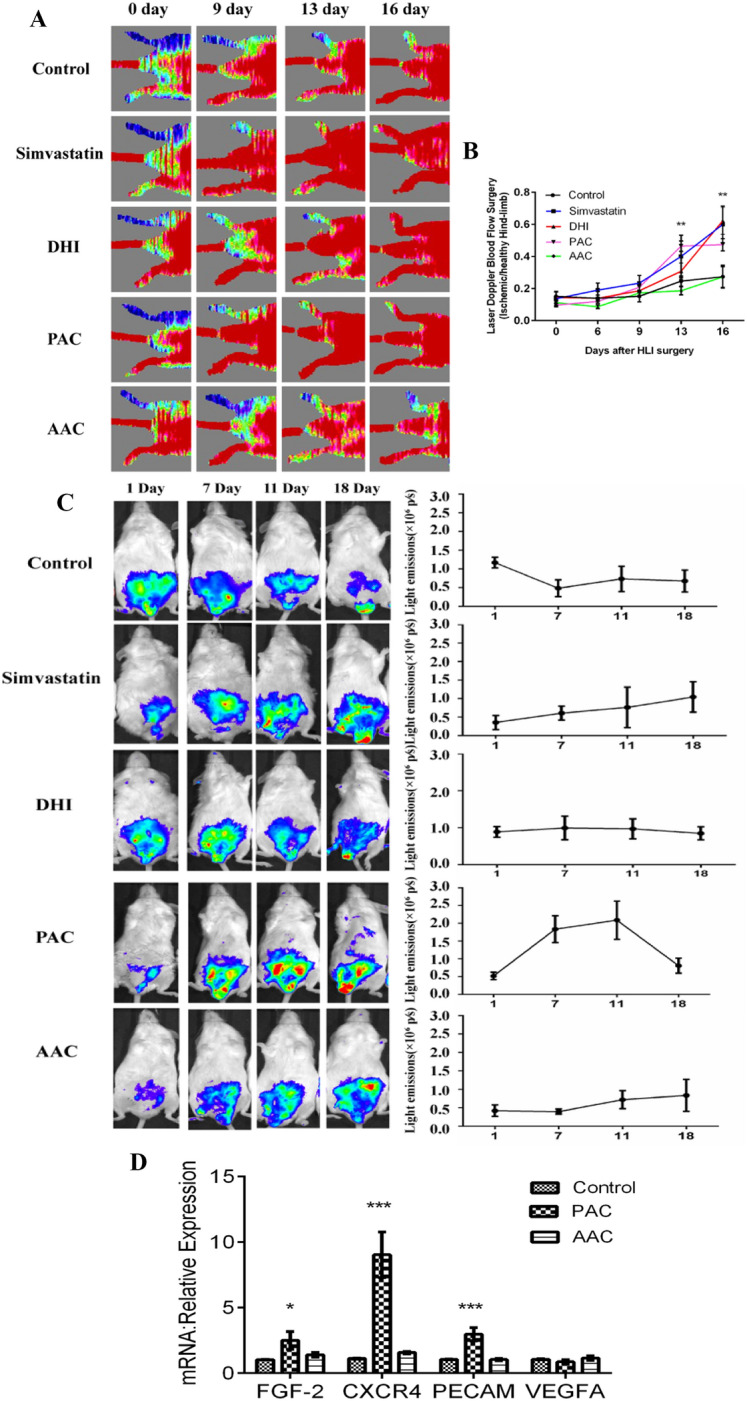
PAC improved perfusion of ischemic limbs and enhanced local angiogenesis. **A** Representative images of laser Doppler perfusion analysis. **B** We calculated the ratio of the ischemic side to the non-ischemic side as the average hindlimb blood flow recovery. PAC significantly improved perfusion recovery on day 13 after HLI surgery. DHI or simvastatin significantly improved perfusion recovery on day 16 after HLI surgery. ^**^P < 0.01 vs. control group. **C** Representative bioluminescent images (left panels) and quantitation (right panels) of VEGFR-2-Luc mice after HLI were obtained at 1, 7, 11, and 18 days in control, simvastatin, DHI, PAC, or AAC-treated groups. Regions of interest (ROI) from displayed images were identified on the HLI sites and quantified as photons per second (p/s). In the hind limb ischemia model, the simvastatin group consist of three mice, and the remaining groups consisted of five mice. **D** Quantitative PCR showed that only PAC increased the expression of FGF-2, CXCR4, and PECAM in ischemic gastrocnemius muscle. ^*^P < 0.05, ^***^P < 0.001 vs. control group. Data are mean ± SEM. We performed the experiments in two copies and repeated them at least three times to ensure reproducibility

Laser Doppler perfusion imaging (LDPI) was performed at different time points (6, 9, 13, and 16 days after hind-limb ischemia surgery). Before surgery, the blood flow ratio between the two hind limbs in all animals was set to 1.0, immediately after induction of hind limb ischemia, the blood flow ratio between ischemic and contralateral normal non-ischemic limbs was 0.128 ± 0.009. Blood flow of saline-treated mice recovered to a ratio of 0.28 ± 0.07 after 16 days, whereas in DHI-, simvastatin-, or PAC-treated mice, the LDPI ratio was accelerated to 0.62 ± 0.09, 0.60 ± 0.11, and 0.44 ± 0.03 respectively after 16 days. In addition, as early as 13 days, PAC-treated mice showed a significantly better recovery of the limb perfusion. Thus, the recovery of severely impaired blood flow in HLI mice was significantly improved by DHI, simvastatin, or PAC treatment. On the other hand, AAC had no such impact in improving the recovery of ischemic limb perfusion (Fig. 5A and B).

To further evaluate the effect of PAC and AAC in an in vivo ischemia-induced angiogenesis, we used VEGFR-2-Luc mice in which the expression of luciferase is driven by VEGFR-2 promoter so that angiogenesis can be directly observed by using bioluminescent imaging techniques.

There was no difference in average bioluminescent intensity between the control group and the treatment group before and after HLI surgery. However, compared to the control group, HLI areas in DHI, simvastatin, or PAC treatment groups showed higher bioluminescent intensities after 7 days, suggesting an up-regulation of VEGFR-2 in the ischemic area and a beginning of accelerated blood-flow perfusion recovery. In contrast, HLI areas in the AAC treatment group showed a higher bioluminescent intensity after 11 days (Fig. 5C). Quantitative PCR was performed to find out whether PAC or AAC could affect the expression of angiogenesis-related genes in HLI mice. A careful look at Fig. 5D showed that the mRNA levels of FGF-2, CXCR4, and PECAM in the PAC group were significantly up-regulated compared with the control, whereas AAC had no effect. Taken together, these results provided further evidence that PAC-treatment promoted recovery of blood flow by increasing the capillary density of the ischemic area.

### AAC, not PAC, reduced Lewis lung carcinoma growth by inhibition of VEGFR2-dependent local angiogenesis

**Fig. 6:**
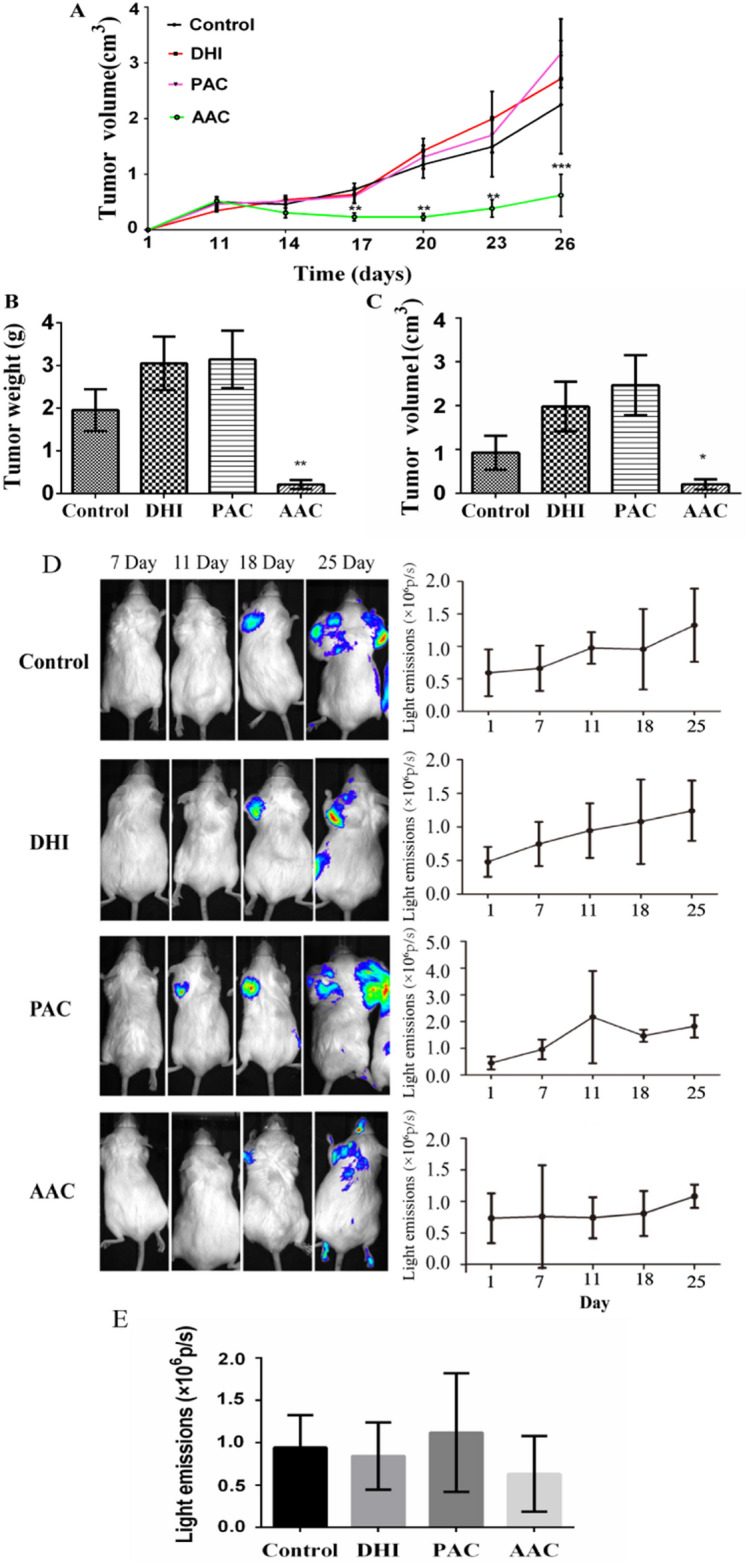
AAC reduced Lewis lung carcinoma growth. Tumor size was continuously monitored from the day of tumor cell transplant up to 26 days. **A** Tumor volume curve of saline, DHI-, PAC-, or AAC-treated mice. **B** Weight of transplanted tumors in each treatment group at day 26. **C** Average tumor volume in each treatment group at day 26. **D** The bioluminescent intensities of the saline, DHI, PAC, or AAC-treated VEGFR2-Luc mice tumor model were dynamically measured. Regions of interest (ROI) from displayed images were identified on the tumor sites and quantified as photons per second (p/s). **E** The bioluminescent intensities of each group on day 25 after treatment. The data displayed are mean ± SEM. Sample number (n ) = 5 in each group. The values ^*^P < 0.05 and ^***^P < 0.001 are significant, compared to the control group

To examine the inhibitory effect of AAC on tumor growth, we transplanted Lewis lung carcinoma cells in VEGFR2-Luc mice and measured the *in-situ* tumor size continually with a caliper for 26 days. The results showed that after 17 days of treatment, the average tumor volumes of the control mice reached 722.89 mm^3^. AAC treatment reduced tumor volume to 232.29 mm^3^. In contrast, PAC treatment had no effect on tumor volume. Though the tumor volumes of AAC-treated mice increased gradually during the 26 day-observation periods, the growth trend was not as fast as the control and PAC groups (Fig. 6C). The above data indicate that AAC strongly retards Lewis lung carcinoma growth in vivo. At the end of the 26 day-treatment, we sacrificed the mice, removed the tumors, and weighed them. The weight and size of tumors in control, DHI, PAC, and AAC-treated groups were shown in Fig. 6B and C. To evaluate the effect of AAC in angiogenesis inhibition, Lewis lung carcinoma cells were transplanted into VEGFR2-Luc mice, where luciferase expression was driven by the VEGFR2 promoter, allowing direct observation of angiogenesis using in vivo bioluminescence intensity. As shown in Fig. 6D, VEGFR2 expression measured in BLI was barely detectable in all groups up to 7 days. However, coincided with the tumor development, bioluminescent intensities increased in control, DHI, and PAC-treated groups 11 days after treatment. In contrast, VEGFR2 expression measured in BLI was significantly delayed in the AAC-treated group (Fig. 6D). After 25 days of treatment, the bioluminescent intensities in the AAC-treated group remained unchanged while the bioluminescent intensities of control, DHI, and PAC-treated groups gradually increased (Fig. 6E). These data suggest that AAC could suppress angiogenesis in vivo.

### Confirmation of bi-directional regulation of angiogenesis with main monomers in PAC and AAC

**Fig. 7:**
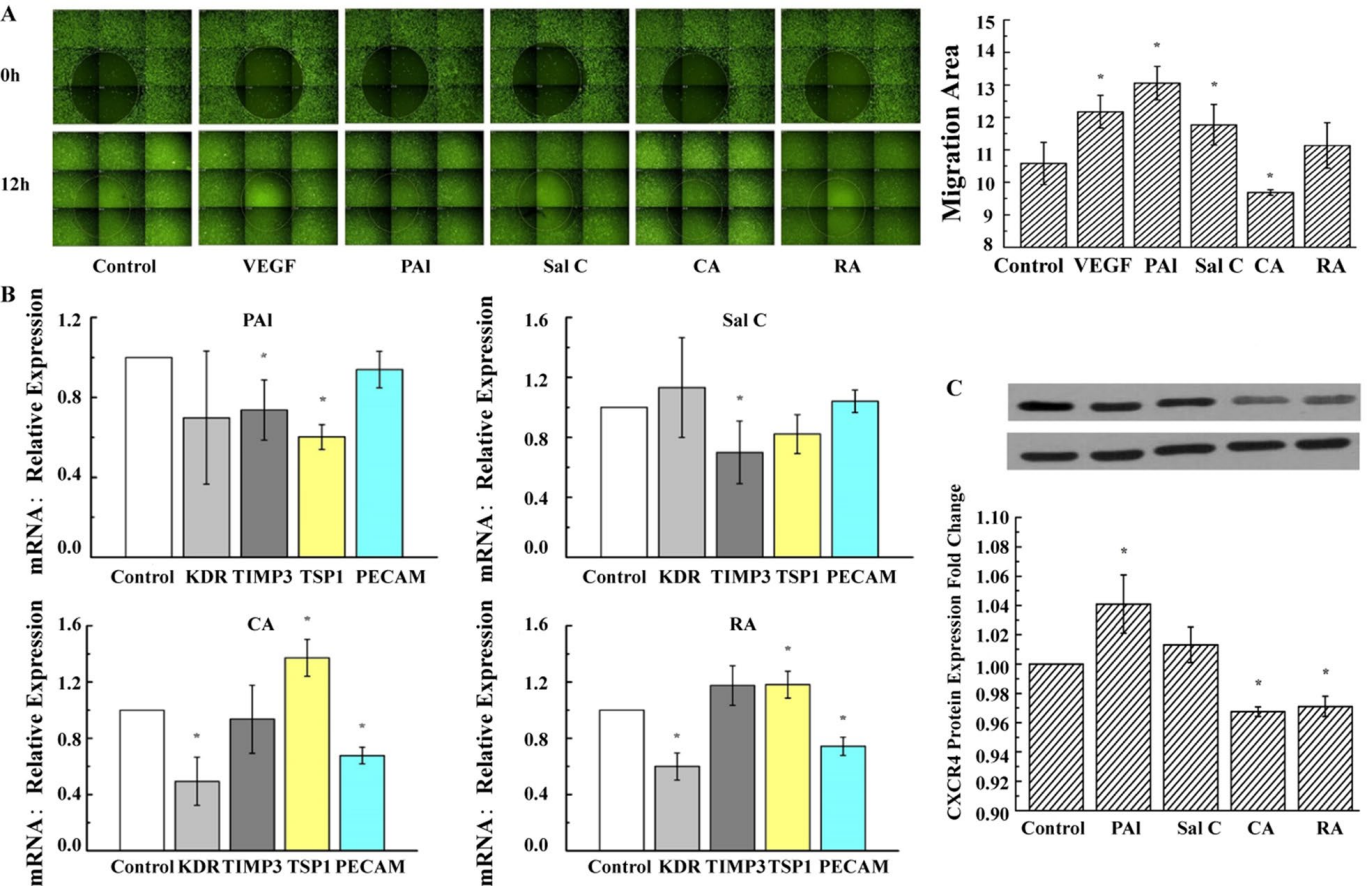
The main monomers of PAC and AAC have a bi-directional regulation of angiogenesis. **A** Representative images of the Oris™ Cell Migration Assay in EA.hy 926 cells. The EC-covered area was analyzed immediately following stopper removal (pre-migration, 0 h) or after drug treatment for 12 h. A detection area (Green Square) was defined in the same location for all wells, and the area covered with cells was determined under the indicated conditions. VEGF, PAl, or Sal C were promoted, while CA was inhibited during the cell migration after 12 h. **B** Quantitative PCR showing the expression of KDR, TIMP3, TSP1, and PECAM-1 in cells treated with PAl, Sal C, CA, or RA in ECs. **C** Representative Western blot analysis of CXCR4 expression (top) and densitometric quantitation (bottom). Compared with control, CXCR4 expression was significantly increased by PAl treatment but significantly decreased by CA and RA treatment. GAPDH was used as a loading control. ^*^P < 0.05 vs. control group. n = 3. Data are presented as mean ± SEM

The migration ability of the main monomers of PAC and AAC in EA.hy926 cells was determined by Oris™ Cell migration assay. Similar to VEGF, PAl and Sal C significantly increased, while CA significantly decreased, migration rate compared with the control group after 12 h (Fig. 7A). To distinguish the molecular mechanisms of (bi-directional regulation) angiogenesis by PAC and AAC, qPCR analyses were performed to find out whether the main monomers of PAC or AAC could affect the expression of angiogenesis-related genes in ECs. As shown in Fig. 7B, PAl decreased the expression of TIMP3 and TSP1; Sal C decreased the expression of TIMP3; CA and RA decreased the expression of KDR and PECAM-1, while increased the expression of TSP1. We used western blotting to independently verify the PAC and AAC effects on angiogenesis-related protein expression. As shown in Fig. 7C, CXCR4 expression was significantly decreased by CA and RA but increased by PAl, consistent with their differential effects on EC migration (Fig. 7A).

## Discussion

We have previously shown that pro-angiogenesis activity of DHI coordinately activated VEGF/VEGFR-2 and PPARδ pathways and accelerated recovery from peripheral arterial disease in type 2 diabetic mice [24]. A defined combination of four active principles from DHI (including tanshinol, protocatechuic aldehyde, salvianolic acid B, and salvianolic acid C) had the same effects to promote tube formation in vitro and improve recovery of ischemic limb perfusion by enhancing angiogenesis in HLI ischemia mice in vivo [25]. We have known some pro-angiogenic components in DHI, but is there any component that anti-angiogenic in DHI at the same time? This study is to further explore the material basis of DHI on angiogenesis regulation. Using reverse pharmacology, DHI chemical fractions were screened by in vitro angiogenesis assay and were found to contain not only a pro-angiogenic component (PAC) but also an anti-angiogenic component (AAC). Subsequently, the pharmacodynamics experiments of PAC and AAC on angiogenesis were carried out in the hind-limb ischemia model and the tumor model, which confirmed that PAC, but not AAC, promoted angiogenesis during hind-limb ischemia while AAC, but not PAC, inhibited angiogenesis in Lewis lung carcinoma implant. To further study the material basis, the active constituents of PAC and AAC were isolated and screened by functional experiments. We found that the DHI active principles PAl and Sal C played a major role in promoting angiogenesis, while CA and RA had the effect of inhibiting angiogenesis.

Angiogenesis is regulated by both activator and inhibitor molecules. Our study provides an example from a single TCM prescription which contains both pro-angiogenic and anti-angiogenic components, a finding of significance to basic biomedical research. Unlike Western medicines that are often monomers, a TCM formular is composed of multiple ingredients acting in a multi-targeted fashion. It is plausible that the bi-directional nature of DHI for angiogenesis allows it to be cardioprotective in a heart disease setting whereas anti-cancer in a tumor setting.

TIMP3 is a tissue inhibitor of metalloproteinases (MMPs), which downregulates the activity of MMPs to degrade the extracellular matrix and inhibit angiogenesis [31, 32]. Angiogenesis inhibitor TSP1, the platelet reactive protein 1, is one of the members of the extracellular matrix protein family, which inhibits the degradation of ECM and affects the migration and survival of endothelial cells [33]. KDR is an endothelial cell growth factor receptor and is a classic angiogenic receptor [34]. PECAM-1 is an endothelial cell adhesion molecule that promotes adhesion between endothelial cells to promote angiogenesis [35]. We found that PAl significantly down-regulated the expression of TIMP3 and TSP1 gene, and Sal C significantly down-regulated the expression of the TIMP3 gene. On the other hand, CA and RA inhibited the expression of KDR and PECAM-1 genes and promoted the expression of the TSP1 gene. Therefore, it can be concluded that the active components in DHI regulate the angiogenesis process mainly by affecting the proliferation, migration, adhesion, and degradation of the extracellular matrix of endothelial cells.

In addition, the active compounds in PAC or AAC can regulate the TSP1 expression, indicating that TSP1 is a common target for the bi-directional regulation of angiogenesis by DHI. In addition, PAl and CA also regulate CXCR4 protein expression in a bi-directional manner. CXCR4 is a specific receptor of chemokine SDF-1, which mediates the migration and homing of hematopoietic stem cells and can promote the secretion of VEGF [36]. As a G protein-coupled receptor, CXCR4 is important for many cellular functions, including migration, mobilization, and homing [37], and plays an important role in enhancing angiogenesis in vivo [38].

One of the challenging tasks of modernization of Chinese herbal medicine is to reveal its complex pharmacological basis and its compatibility mechanism [39]. Attentions on effective combinatorial mixtures are increasing in research of drug discovery and development [40]. Further development of DHI will be based on the traditional Chinese prescription compatibility theory, guided by systematic scientific thoughts to understand the effective combination of the components for different disease treatments. Combination components exploit the chances for better efficacy and decreased toxicity [41]. It is an effective method for developing new modern drugs based on the combination of effective monomers in TCM with exact clinical efficacy.

This present work only examined four monomers (PAl, Sal C, CA, and RA) individually, but the overall impact of PAC or AAC is likely contributed by combinations of multiple components. Therefore, further experiments are needed to clarify the optimal monomer combination that promotes or inhibits angiogenesis. It is also possible that other unidentified compounds exist that potentially contribute to promoting or inhibiting angiogenesis. In DHI, a total of sixty-three compounds have been identified and characterized so far [18]. Other monomers in PAC and AAC that promote or inhibit angiogenesis remain to be identified.

Many pro-angiogenic and anti-angiogenic molecules have been identified, and some have been developed into drugs to treat cardiovascular disease and cancer, respectively. However, we showed that the widely used cardiovascular herbal remedy, DHI, has intrinsic pro- and anti-angiogenesis dual activities. Moreover, the bidirectional activities are defined by different yet overlapping combinations of multiple molecules in DHI.

Finally, our finding is of clinical significance from the perspective of precision medicine and safety pharmacology. For example, despite of its proven efficacy for a variety of cardiovascular diseases, is it safe and effective for certain cancer patients or patients at risk of cancer? Is it possible to develop DHI-based precision Chinese medicine that target only pro- or anti-angiogenesis process? Future basic science research and clinical trials are required to fully answer these questions.

## Conclusions

We conclude that DHI, a widely used compound Chinese patent medicine, contained both pro-and anti-angiogenesis components that compose different chemical combinations. The pro-angiogenesis component can be capable of promoting ischemic vascular injury repair, whereas the anti-angiogenesis components inhibited tumor growth. A key mechanism involved in the promotion of angiogenesis is associated with PAl-mediated TIMP and TSP1 downregulation and CXCR4 upregulation, as well as Sal C-mediated TIMP3 downregulation. We showed for the first time that the mechanism of DHI’s inhibition of angiogenesis is at least partly related to the down-regulation of KDR, PECAM-1 and CXCR4 by CA/RA.

## Data Availability

The datasets during and/or analyzed during the current study are available from the corresponding author on reasonable request.

## Abbreviations

HLI: Hind-limb ischemia
BLI: Bioluminescence imaging
PAC: Pro-angiogenic components
AAC: Anti-angiogenic components
5-HMF: 5-hydroxymethyl-2-furfural
DSS: Danshensu
PAc: Protocatechuic acid
PAl: Protocatechuic aldehyde
HSYA: Hydroxysafflor yellow A
CA: Caffeic acid
RA: Rosmarinic acid
LA: Lithospermic acid
Sal B: Salvianolic acid B
Sal A: Salvianolic acid A
Sal C: Salvianolic acid C

## References

[1] Rivera LB, Bergers G (2014). Angiogenesis. Targeting vascular sprouts. Science.

[2] Shah AM, Mann DL (2011). In search of new therapeutic targets and strategies for heart failure: recent advances in basic science. Lancet.

[3] van der Laan AM, Piek JJ, van Royen N (2009). Targeting angiogenesis to restore the microcirculation after reperfused MI. Nat Rev Cardiol.

[4] Yi M, Jiao D, Qin S, Chu Q, Wu K, Li A (2019). Synergistic effect of immune checkpoint blockade and anti-angiogenesis in cancer treatment. Mol Cancer.

[5] Albini A, Tosetti F, Benelli R, Noonan DM (2005). Tumor inflammatory angiogenesis and its chemoprevention. Cancer Res.

[6] Qin S, Li A, Yi M, Yu S, Zhang M, Wu K (2019). Recent advances on anti-angiogenesis receptor tyrosine kinase inhibitors in cancer therapy. J Hematol Oncol.

[7] Sengupta S, Toh SA, Sellers LA, Skepper JN, Koolwijk P, Leung HW (2004). Modulating angiogenesis: the yin and the yang in ginseng. Circulation.

[8] Liu HX, Wang Y, Lu Q, Yang MZ, Fan GW, Karas RH (2016). Bidirectional regulation of angiogenesis by phytoestrogens through estrogen receptor-mediated signaling networks. Chin J Nat Med.

[9] Chen RG, Xu H, He Y, Zhu S (2013). Research progress on pro- or anti-angiogenic effects of Chinese materia medica fomulas and their active components. Chin Trad Herb Drugs.

[10] Cao W, Hu C, Wu L, Xu L, Jiang W (2016). Rosmarinic acid inhibits inflammation and angiogenesis of hepatocellular carcinoma by suppression of NF-kappaB signaling in H22 tumor-bearing mice. J Pharmacol Sci.

[11] Feng X, Li Y, Wang Y, Li L, Little PJ, Xu SW (2019). Danhong injection in cardiovascular and cerebrovascular diseases: pharmacological actions, molecular mechanisms, and therapeutic potential. Pharmacol Res.

[12] Orgah JO, He S, Wang Y, Jiang M, Wang Y, Orgah EA (2020). Pharmacological potential of the combination of Salvia miltiorrhiza (Danshen) and Carthamus tinctorius (Honghua) for diabetes mellitus and its cardiovascular complications. Pharmacol Res.

[13] Lyu M, Yan CL, Liu HX, Wang TY, Shi XH, Liu JP (2017). Network pharmacology exploration reveals endothelial inflammation as a common mechanism for stroke and coronary artery disease treatment of Danhong injection. Sci Rep.

[14] Chen Y, Liu M, Zhao T, Zhao B, Jia L, Zhu Y (2014). Danhong injection inhibits the development of atherosclerosis in both Apoe(-)/(-) and Ldlr(-)/(-) mice. J Cardiovasc Pharmacol.

[15] Liu M, Pan Q, Chen Y, Yang X, Zhao B, Jia L (2015). Administration of Danhong Injection to diabetic db/db mice inhibits the development of diabetic retinopathy and nephropathy. Sci Rep.

[16] Owoicho Orgah J, Wang M, Yang X, Wang Z, Wang D, Zhang Q (2018). Danhong injection protects against hypertension-induced renal injury via down-regulation of myoglobin expression in spontaneously hypertensive rats. Kidney Blood Press Res.

[17] Jiang M, Jiao Y, Wang Y, Xu L, Wang M, Zhao B (2014). Quantitative profiling of polar metabolites in herbal medicine injections for multivariate statistical evaluation based on independence principal component analysis. PloS ONE.

[18] Zhang QQ, Dong X, Liu XG, Gao W, Li P, Yang H (2016). Rapid separation and identification of multiple constituents in Danhong Injection by ultra-high performance liquid chromatography coupled to electrospray ionization quadrupole time-of-flight tandem mass spectrometry. Chin J Nat Med.

[19] Wang D, Fan G, Wang Y, Liu H, Wang B, Dong J (2013). Vascular reactivity screen of Chinese medicine danhong injection identifies Danshensu as a NO-independent but PGI2-mediated relaxation factor. J Cardiovasc Pharmacol.

[20] Yang X, Orgah J, Wang D, Fan G, Jingyang H, Han J (2017). Danhong injection reduces vascular remodeling and up-regulates the Kallikrein-kinin system in spontaneously hypertensive rats. Sci Rep.

[21] Yang M, Orgah J, Zhu J, Fan G, Han J, Wang X (2016). Danhong injection attenuates cardiac injury induced by ischemic and reperfused neuronal cells through regulating arginine vasopressin expression and secretion. Brain Res.

[22] Orgah JO, Ren J, Liu X, Orgah EA, Gao XM, Zhu Y (2019). Danhong injection facilitates recovery of post-stroke motion deficit via Parkin-enhanced mitochondrial function. Restor Neurol Neurosci.

[23] Orgah JO, Yu J, Zhao T, Wang L, Yang M, Zhang Y (2018). Danhong injection reversed cardiac abnormality in brain-heart syndrome via local and remote beta-adrenergic receptor signaling. Front Pharmacol.

[24] He S, Zhao T, Guo H, Meng Y, Qin G, Goukassian DA (2016). Coordinated activation of VEGF/VEGFR-2 and PPARdelta pathways by a multi-component chinese medicine DHI accelerated recovery from peripheral arterial disease in type 2 diabetic mice. PLoS ONE.

[25] He S, Guo H, Zhao T, Meng Y, Chen R, Ren J (2019). A defined combination of four active principles from the danhong injection is necessary and sufficient to accelerate EPC-mediated vascular repair and local angiogenesis. Front Pharmacol.

[26] Chen J, Cao W, Asare PF, Lv M, Zhu Y, Li L (2016). Amelioration of cardiac dysfunction and ventricular remodeling after myocardial infarction by danhong injection are critically contributed by anti-TGF-beta-mediated fibrosis and angiogenesis mechanisms. J Ethnopharmacol.

[27] Zhao T, Chang L, Zhang B, Lu M, Wang X, Orgah JO (2017). Specific combination of salvianolic acids as core active ingredients of danhong injection for treatment of arterial thrombosis and its derived dry gangrene. Front Pharmacol.

[28] Fu J, Yu J, Chen J, Xu H, Luo Y, Lu H (2018). In vitro inhibitory properties of sesquiterpenes from Chloranthus serratus on cell motility via down-regulation of LIMK1 activation in human breast cancer. Phytomedicine.

[29] Michaud SE, Menard C, Guy LG, Gennaro G, Rivard A (2003). Inhibition of hypoxia-induced angiogenesis by cigarette smoke exposure: impairment of the HIF-1alpha/VEGF pathway. FASEB J.

[30] Zhang N, Fang Z, Contag PR, Purchio AF, West DB (2004). Tracking angiogenesis induced by skin wounding and contact hypersensitivity using a Vegfr2-luciferase transgenic mouse. Blood.

[31] Basu R, Lee J, Morton JS, Takawale A, Fan D, Kandalam V (2013). TIMP3 is the primary TIMP to regulate agonist-induced vascular remodelling and hypertension. Cardiovasc Res.

[32] Qi JH, Anand-Apte B (2015). Tissue inhibitor of metalloproteinase-3 (TIMP3) promotes endothelial apoptosis via a caspase-independent mechanism. Apoptosis.

[33] Bienes-Martinez R, Ordonez A, Feijoo-Cuaresma M, Corral-Escariz M, Mateo G, Stenina O (2012). Autocrine stimulation of clear-cell renal carcinoma cell migration in hypoxia via HIF-independent suppression of thrombospondin-1. Sci Rep.

[34] Carmeliet P (2000). Mechanisms of angiogenesis and arteriogenesis. Nat Med.

[35] Park S, Sorenson CM, Sheibani N (2015). PECAM-1 isoforms, eNOS and endoglin axis in regulation of angiogenesis. Clin Sci.

[36] Kawakami Y, Ii M, Matsumoto T, Kuroda R, Kuroda T, Kwon SM (2015). SDF-1/CXCR4 axis in Tie2-lineage cells including endothelial progenitor cells contributes to bone fracture healing. J Bone Miner Res.

[37] Yamaguchi J, Kusano KF, Masuo O, Kawamoto A, Silver M, Murasawa S (2003). Stromal cell-derived factor-1 effects on ex vivo expanded endothelial progenitor cell recruitment for ischemic neovascularization. Circulation.

[38] Chen L, Wu F, Xia WH, Zhang YY, Xu SY, Cheng F (2010). CXCR4 gene transfer contributes to in vivo reendothelialization capacity of endothelial progenitor cells. Cardiovasc Res.

[39] Li F, Fan XX, Chu C, Zhang Y, Kou JP, Yu BY (2018). A strategy for optimizing the combination of active components based on chinese medicinal formula Sheng-Mai-San for myocardial ischemia. Cell Physiol Biochem.

[40] Lu JJ, Pan W, Hu YJ, Wang YT (2012). Multi-target drugs: the trend of drug research and development. PloS ONE.

[41] Foucquier J, Guedj M (2015). Analysis of drug combinations: current methodological landscape. Pharmacol Res Perspect.

